# Adaptability Protects University Students From Anxiety, Depression, and Insomnia During Remote Learning: A Three-Wave Longitudinal Study From China

**DOI:** 10.3389/fpsyt.2022.868072

**Published:** 2022-04-18

**Authors:** Keshun Zhang, Zhenhong Mi, Elizabeth J. Parks-Stamm, Wanjun Cao, Yaqi Ji, Runjie Jiang

**Affiliations:** ^1^Department of Psychology, Normal College, Qingdao University, Qingdao, China; ^2^Student Counselling and Mental Health Center, Qingdao University, Qingdao, China; ^3^Department of Psychology, University of Southern Maine, Portland, ME, United States; ^4^UCL Queen Square Institute of Neurology, University College London, London, United Kingdom

**Keywords:** COVID-19, remote learning, adaptability, insomnia, anxiety, depression

## Abstract

The longitudinal relationship between students’ pre-existing adaptability and subsequent sleep and mental health during the COVID-19 pandemic has not been studied. The present study examines the relationship between adaptability and students’ anxiety, depression, and insomnia during and after the lockdown related to COVID-19. 5,235 university students participated in a longitudinal study with three time points. Students completed the Adaptability Scale before the outbreak (October 2019; Time 1), the Insomnia Severity Index (ISI) both during (April 2020; Time 2) and after lockdown (March 2021; Time 3), the Anxiety and Depression subscales of the SCL-90 (at Time 1 and 3), and the SAS/SDS (at Time 2). The results showed that self-reported adaptability is significantly negatively correlated with anxiety and depression, and that anxiety and depression are positively correlated with insomnia. Furthermore, adaptability protects from insomnia both directly and through its negative relationship with anxiety and depression. This study sheds light on the internal mechanisms mediating the relationship between students’ adaptability and experience of insomnia in challenging circumstances. Implications for curtailing the negative effects of stressful events on students’ sleep health by improving their adaptability and reducing their anxiety and depression are discussed.

## Introduction

Numerous studies have demonstrated the relationship between stressful events—like natural disasters—and negative outcomes like insomnia (1), anxiety (2), and depression (3). Unlike localized natural disasters (e.g., flooding, earthquakes), the COVID-19 pandemic was a natural disaster that impacted individuals around the world (4), with a disproportionate impact on university students (5).

**FIGURE 1:**
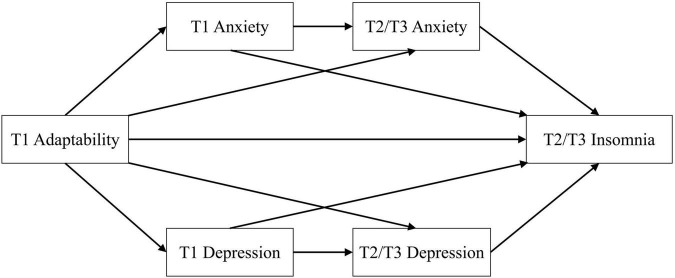
Conceptual model of adaptability, anxiety, depression, and insomnia.

As universities shut down and students suddenly were removed from their academic and social supports (6), university students reported declines in their physical and mental health (7–11). In line with previous research documenting sleep disturbances in response to traumatic events (12), insomnia has been identified as an important adverse outcome of the COVID-19 pandemic (13, 14), particularly for university students (5). Insomnia is defined as a difficulty with sleep onset and sleep maintenance resulting in impairment and distress (15). Adaptability has emerged as an important protective factor for individuals in the pandemic (16–19). The present study therefore examines the long-term impact of adaptability—measured before the pandemic—on the development of insomnia in university students both during and after lockdown, along with potential mediators of this relationship (i.e., anxiety and depression; see Figure 1).

Adaptability is defined as the ability to adjust one’s cognition, emotion, and behavior in response to changing, new, or uncertain conditions (20, 21). During the COVID-19 pandemic, students needed to adjust to vast changes to their daily lives associated with the closure of universities, confinement to one’s home, and the shift to remote learning. Individuals with greater adaptability showed more positive academic emotions and greater academic engagement in remote learning (19). In addition, adaptability predicted their distress and mood during COVID-19, controlling for personality, mattering, and automatic thoughts (16).

Adaptability thus appears to be a beneficial predispositional factor for students. The 3P model of insomnia (also known as the Spielman model) is a widely used theoretical framework for explaining insomnia (22–24). According to the 3P model of insomnia, persistent insomnia can be explained by a combination of three factors, namely predisposing factors, precipitating factors, and perpetuating factors (25). Predisposing factors include characteristics that predispose an individual to insomnia, such as genetics, cognitive style, awakening ability, or the tendency to ruminate or worry (26, 27). However, predisposing factors can also be beneficial factors, such as adaptability or mindfulness, that negatively predict insomnia. Precipitating factors include factors and events that trigger sudden insomnia, such as stressful events, or environmental or psychological stressors (25). Perpetuating factors include maladaptive emotions, such as sleep-related anxiety (28) or coping behaviors, such as going to bed early, that the individual enacts to cope with sleep disturbances. Predisposing factors and precipitating factors interact to cause temporary sleep disturbances, whereas maintenance factors make the individual’s insomnia symptoms persist (26). The present study examines the predisposing factor of adaptability (and its effect on other predisposing factors like anxiety and depression) in the context of the precipitating factors of the pandemic and the shift to remote learning.

Past research on the key role of adaptability as a protective factor for insomnia following natural disasters suggests that adaptability may play a beneficial role as a predisposing factor in individuals’ sleep health during the COVID-19 pandemic. Varela et al. (29), for example, found adaptability was the only significant predictor of insomnia following an earthquake in Greece. Liu et al. (30) found that the hardiness factor of psychological resilience (i.e., the ability to adapt to adversity) was a protective factor for insomnia among medical professionals working in the COVID-19 response in China.

Given the relationship between stressful events and insomnia (31, 32), it not surprising that insomnia and other sleep complaints increased during the precipitating event of the COVID-19 pandemic. According to the NHS (33), insomnia refers to difficulty in going to sleep, waking up during the night, waking up early, feeling irritable during the day, and feeling tired after waking up on a regular basis. In France, the prevalence of sleep problems increased from 49% before the pandemic to 74% during the lockdown (34). Similarly, Lin et al. (35) reported a 37% increase in clinical insomnia in China from before to during the pandemic. However, individual variability in sleep responses to the pandemic are striking. In one study, whereas approximately 29% of participants showed a decrease in their sleep quality over the first weeks of the pandemic, approximately 47% showed an improvement in their sleep (36). We propose that individuals’ adaptability—and the effect of adaptability on their psychological well-being—may explain this variability in sleep outcomes across individuals in the year following the outbreak of COVID-19.

How does adaptability impact individuals’ sleep in times of crisis? We propose that adaptability impacts individuals’ levels of anxiety and depression, which then impacts their sleep (see Figure 1). Previous researchers have proposed that stress—like that experienced during the COVID-19 pandemic– induces maladaptive responses like anxiety, which in turn affect sleep quality (26, 37). The pandemic has been associated with a significant increase in anxiety and depression around the world [e.g., a meta-analysis from (38) estimated an increase of 31.9 and 33.7%, respectively] and among university students in China in particular (39). Adaptability, on the other hand, plays a protective role by buffering individuals from these negative emotional responses to challenging circumstances. University students high in adaptability, for example, reported fewer negative emotions during the pandemic, including anxiety, hopelessness, and boredom (19).

Concerning the second step of this model (see Figure 1), many studies have demonstrated the relationship between both the predisposing factors of depression and anxiety and insomnia (40–42). Further, both anxiety and depression have been found to mediate the relationship between other factors and insomnia; for example, anxiety and depression mediate the relationship between conscientiousness/stability and insomnia (43), and anxiety mediated the relationship between perfectionism and insomnia in a longitudinal study (44). This research suggests that anxiety and depression play a proximal role in insomnia.

However, a good deal of research has indicated reciprocal relationships between anxiety, depression, and insomnia (42, 45), indicating the need to measure changes over time. Previous studies have mostly relied on cross-sectional designs to examine insomnia during the COVID-19 pandemic [e.g., (8, 46–48)], which are unable to examine relationships between pre-pandemic individual characteristics and subsequent mental and sleep health over time during the pandemic. Our longitudinal design addresses this gap in the research.

In sum, our study has two aims. The first aim is to explore the relationship between adaptability as a predisposing individual difference and insomnia both during and after the lockdowns associated with COVID-19. The second aim is to understand the mediating role of anxiety and depression in this relationship. Using a longitudinal design, we first examine if adaptability measured before the pandemic influences insomnia during and after the lockdown. Second, we examine whether anxiety and depression (measured at Time 2 and 3) influence sleep quality both during and after the lockdown, that is, the possible mediating role of anxiety and depression in the relationship between adaptability and insomnia.

## Materials and Methods

### Ethics Statement

This study was approved by our university’s Research Ethics Committee. All procedures complied with the ethical standards of the latest version of the Helsinki Declaration. All participants willingly gave their informed consent digitally after being informed about the purpose of the study. All analyses were based on anonymous data.

### Participants and Design

Longitudinal data was collected via a Chinese online research panel, Wenjuanxing^1^, which is functionally equivalent to Amazon Mechanical Turk. 8,547 university students participated in the Time 1 (T1) assessment. Of these, 5,408 took part in the Time 2 (T2) assessment and 5,235 took part in the Time 3 (T3) assessment (2,747 females, 2,488 males), with a 38.75% attrition rate. There were no sample differences among the three waves in age (*F* = 1.11, *p* = 0.33) or gender (Kruskal–Wallis test; χ = 2.20, *p* = 0.33) (49). The final sample size was 5,235, with mean age = 19.00 years, SD = 0.89.

T1 took place in October 2019 (before the COVID-related restrictions) and measured adaptability, anxiety, and depression. T2 took place in April 2020 (during the COVID-related restrictions and remote learning) and measured anxiety, depression, and insomnia. T3 took place in March 2021 (after the COVID-related restrictions were lifted) and measured anxiety, depression, and insomnia.

### Measures

#### Adaptability Scale

Students’ adaptability was assessed using the nine-item Adaptability Scale [(50); Chinese version: (19)] in T1. This scale comprises six items referring to cognitive-behavioral adaptability (e.g., “I am able to think through a number of possible options to assist me in a new situation”) and three items referring to affective adaptability (e.g., “When uncertainty arises, I am able to minimize frustration or irritability so that I can deal with it best”). The items were assessed on a 7-point scale ranging from 1 (Strongly disagree) to 7 (Strongly agree). The scale showed good internal consistency (α_*t*1_ = 0.91).

#### Anxiety and Depression Subscales of the Symptom Checklist 90

We applied the anxiety and depression subscales of the SCL-90 (51) to assess anxiety and depression at both T1 and T3. The anxiety and depression subscales include 10 items and 13 items, respectively. The items were rated along a 5-point response scale with 1–5 representing the severity as follows: “1 = none,” “2 = light,” “3 = moderate,” “4 = quite heavy,” and “5 = severe.” The SCL-90 uses a standardized scoring algorithm to define anxiety symptoms, with a total score range of 10–50. Individuals were categorized as experiencing anxiety symptoms if the anxiety subscale score was >20. A standardized scoring algorithm is also used to define depression symptoms, with a total score range of 13–65. Individuals were categorized as experiencing depression symptoms if the anxiety subscale score was >26. The anxiety and depression subscales were internally consistent (α_*t*1_ = 0.85 and 0.89, respectively; α_*t*3_ = 0.89 and 0.92, respectively).

#### Zung Self-Rating Anxiety Scale

Anxiety at T2 was measured by the SAS [(52); Chinese version: (53)], which contains 20 items. The scale covers both psychological symptoms (e.g., “I feel afraid for no reason at all”) and somatic symptoms (e.g., “My arms and legs shake and tremble”). The items were rated along a 4-point response scale ranging from 1 (A little of the time) to 4 (Most of the time). The SAS uses a standardized scoring algorithm to define anxiety symptoms, with a total score range of 25–100. Individuals were categorized as experiencing anxiety symptoms if the SAS score was greater than or equal to 50. The scale was internally consistent (α_*t*2_ = 0.76).

#### Zung Self-Rating Depression Scale

Depression at T2 was evaluated by the SDS [(54); Chinese version: (55)]. The scale contains 20 items, of which 10 items indicate negative experience (e.g., “I feel unhappy and depressed”) and 10 items indicate positive experience (e.g., “I am hopeful for the future”; reverse coded). Participants responded using a 4-point Likert scale ranging from 1 (A little of the time) to 4 (Most of the time). The SDS uses a standardized scoring algorithm to define depression symptoms, with a total score range of 25–100. Individuals were categorized as experiencing depression symptoms if the SDS score was greater than or equal to 50. The scale was internally consistent (α_*t*2_ = 0.86).

#### Insomnia Severity Index

The ISI (15) was used to measure the severity of insomnia during COVID-19 at both T2 and T3. This scale includes 7 items that are rated on a 0–4 scale. The total score ranges from 0 to 28, with a higher score indicating a greater number of insomnia symptoms (0–7 = No clinically significant insomnia; 8–14 = Subthreshold insomnia; 15–21 = Moderate severity clinical insomnia; 22–28 = Severe clinical insomnia). The scale was internally consistent (α_*t*2_ = 0.88; α_*t*3_ = 0.88).

### Data Analysis

The statistical analyses were performed using SPSS Version 25.0, with the PROCESS macro for SPSS utilized for the multiple mediation model (56). PROCESS Model 82 was used to test the mediating role of anxiety and depression (mediators) in the relationship between adaptability at T1 (independent variable) and insomnia at T2 (dependent variable Model 1) and T3 (dependent variable in Model 2). 5,000 bootstrap samples and the 95% bias-corrected confidence interval (95% CI) were set to examine the significance of the two mediation effects (56). As different scales for anxiety and insomnia were used at different time points, scores were standardized to allow comparison. The statistical significance level was set at *p* < 0.05.

### Common Method Biases

The Harman single-factor test was used to diagnose the common method bias (57). The results of principal component factor analysis without rotation showed that there were 17 factors whose eigenvalues were greater than 1. The variance explained by the first factor was 19.73%, which falls below the threshold of 40%. The results showed that there is no serious common method bias in this study.

## Results

### Descriptive Statistics and Correlations

The means (*M*), standard deviations (*SD*), skewness, and kurtosis, and reliabilities for all variables across the two-time points are displayed in Table 1. All the measures had acceptable reliabilities (ranging from 0.76 to 0.92).

**TABLE 1:** Descriptive statistics of all study variables (*n* = 5235).

	*M*	*SD*	Skewness	Kurtosis	α
1. T1 Adaptability	5.95	0.82	−1.20	2.90	0.91
2. T1 Anxiety	1.44	0.49	1.94	5.14	0.85
3. T1 Depression	1.44	0.52	1.86	4.38	0.89
4. T2 Anxiety	1.44	0.30	1.06	2.14	0.76
5. T2 Depression	1.69	0.43	0.66	−0.24	0.86
6. T3 Anxiety	1.26	0.42	2.85	11.15	0.89
7. T3 Depression	1.38	0.51	1.88	3.93	0.92
8. T2 Insomnia	1.36	0.48	2.16	6.48	0.88
9. T3 Insomnia	1.44	0.50	1.91	5.25	0.88

*T1 Adaptability (range from 1 to 7), T2 Anxiety and T2 Depression (range from 1 to 4), T1 Anxiety, T1 Depression, T3 Anxiety, and T3 Depression (range from 1 to 5), T2 Insomnia and T3 Insomnia (range from 0 to 4).*

Pearson correlations for the relations between the variables are displayed in Table 2. T1 adaptability was negatively correlated with T1, T2, and T3 anxiety (*rs* = −0.32, −0.15, and −0.14, respectively; *ps* < 0.01); T1, T2, and T3 depression (*rs* = −0.36, −0.19, and −0.16, respectively; *ps* < 0.01); and T2 and T3 insomnia (*rs* = −0.13, −0.14, respectively; *ps* < 0.01). Moreover, T2 insomnia was positively correlated with T1, T2, and T3 anxiety (*rs* = 0.28, 0.49, and 0.30, respectively; *ps* < 0.01), and T1, T2, and T3 depression (*rs* = 0.30, 0.40, and 0.31, respectively; *ps* < 0.01). In addition, T3 insomnia was positively correlated with T1, T2, and T3 anxiety (*rs* = 0.27, 0.31, and 0.56, respectively; *ps* < 0.01), and T1, T2, and T3 depression (*rs* = 0.28, 0.28, and 0.56, respectively; *ps* < 0.01).

**TABLE 2:** Pearson correlation of all study variables (*n* = 5235).

	1	2	3	4	5	6	7	8	9
1. T1 Adaptability	–								
2. T1 Anxiety	−0.32**	–							
3. T1 Depression	−0.36**	0.83**	–						
4. T2 Anxiety	−0.15**	0.27**	0.30**	–					
5. T2 Depression	−0.19**	0.26**	0.32**	0.74**	–				
6. T3 Anxiety	−0.14**	0.37**	0.35**	0.33**	0.30**	–			
7. T3 Depression	−0.16**	0.35**	0.41**	0.34**	0.35**	0.76**	–		
8. T2 Insomnia	−0.13**	0.28**	0.30**	0.49**	0.40**	0.30**	0.31**	–	
9. T3 Insomnia	−0.14**	0.27**	0.28**	0.31**	0.28**	0.56**	0.56**	0.38**	–

**p < 0.01.

### The Multiple Mediation Effects of Adaptability, Anxiety, Depression, and Insomnia at T1 and T2

**FIGURE 2:**
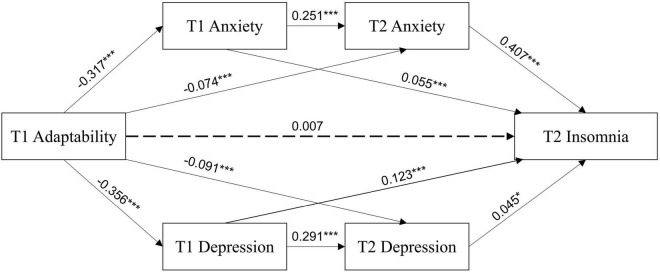
Multiple-mediating test of adaptability, anxiety, depression, and insomnia at T1 and T2. *p < 0.05, ***p < 0.001.

The results of the regression analysis are shown in Figure 2 and Table 3. To first review the role of T1 adaptability: T1 adaptability significantly negatively predicted T1 anxiety (β = −0.317, *p* < 0.001), T2 anxiety (β = −0.074, *p* < 0.001), T1 depression (β = −0.356, *p* < 0.001), and T2 depression (β = −0.091, *p* < 0.001). However, T1 adaptability did not significantly predict T2 insomnia (β = 0.007, *p* = 0.558).

**TABLE 3:** Regression analysis of variable relationships at T1 and T2 (*n* = 5235).

Dependent variables	Predictors	Model summary				
		*F*	*R* ^2^	β	*SE*	*t*
T1 Anxiety	T1 Adaptability	584.13***	0.10	–0.317	0.016	–24.17
T2 Anxiety	T1 Adaptability	228.07***	0.08	–0.074	0.017	–5.29
	T1 Anxiety			0.251	0.014	17.95
T1 Depression	T1 Adaptability	758.14***	0.13	–0.356	0.016	–27.53
T2 Depression	T1 Adaptability	328.68***	0.11	–0.091	0.017	–6.50
	T1 Depression			0.291	0.014	20.87
T2 Insomnia	T1 Adaptability	386.26***	0.27	0.007	0.007	0.59
	T1 Anxiety			0.055	0.010	2.59
	T2 Anxiety			0.407	0.009	22.94
	T1 Depression			0.123	0.010	5.67
	T2 Depression			0.045	0.009	2.50

***p < 0.001.

T1 anxiety and depression also had significant effects. T1 anxiety positively predicted T2 anxiety (β = 0.251, *p* < 0.001) and T2 insomnia (β = 0.055, *p* < 0.001). T1 depression positively predicted T2 depression (β = 0.291, *p* < 0.001) and T2 insomnia (β = 0.123, *p* < 0.001). T2 depression positively predicted T2 insomnia (β = 0.045, *p* < 0.05). A strong regressive path was shown between T2 anxiety and T2 insomnia (β = 0.407, *p* < 0.001).

The final regression model for insomnia, which included T1 adaptability, T1 anxiety, T2 anxiety, T1 depression, and T2 depression, was highly significant and accounted for 27% of the variability in T2 insomnia.

The bootstrap method was used to sample 5,000 times and build a 95% unbiased correction confidence interval for our multiple-mediating model for T1 and T2. The direct path from T1 adaptability to T2 insomnia was not significant (β = 0.007, 95% CI [−0.010, 0.019]). The chain intermediary effect of T1 and T2 anxiety (β = −0.032, 95% CI [−0.039, −0.026]) was significant, as well as the chain intermediary effect of T1 and T2 depression (β = −0.005, 95% CI [−0.009, −0.000]), indicating a significant mediation by both anxiety and depression at two time points. These two paths accounted for 23.02 and 3.60% of the total effect, respectively. T1 adaptability also had indirect effects on T2 insomnia through T1 anxiety (β = −0.017, 95% CI [−0.033, −0.002]), T2 anxiety (β = −0.030, 95% CI [−0.043, −0.018]), T1 depression (β = −0.044, 95% CI [−0.063, −0.026]), and T2 depression (β = −0.004, 95% CI [−0.009, −0.000]), which accounted for 12.23, 21.58, 31.65, and 2.88% of the total effect, respectively (see Table 4).

**TABLE 4:** Mediation effects test at T1 and T2.

	Indirect paths (Ind)	Indirect effect			Percentage
		Effect	95% confidence interval		
			BootLLCI	BootULCI	
Ind 1	T1 Adaptability→T1 Anxiety→T2 Insomnia	−0.017	−0.033	−0.002	12.23%
Ind 2	T1 Adaptability→T2 Anxiety→T2 Insomnia	−0.030	−0.043	−0.018	21.58%
Ind 3	T1 Adaptability→T1 Depression→T2 Insomnia	−0.044	−0.063	−0.026	31.65%
Ind 4	T1 Adaptability→T2 Depression→T2 Insomnia	−0.004	−0.009	−0.000	2.88%
Ind 5	T1 Adaptability→T1 Anxiety→T2 Anxiety→T2 Insomnia	−0.032	−0.039	−0.026	23.02%
Ind 6	T1 Adaptability→T1 Depression→T2 Depression→T2 Insomnia	−0.005	−0.009	−0.000	3.60%
Sum		−0.132	−0.186	−0.138	94.96%

*Percentage, Percentage of total effect explained.*

### The Multiple Mediation Effects of Adaptability, Anxiety, Depression, and Insomnia at T1 and T3

**FIGURE 3:**
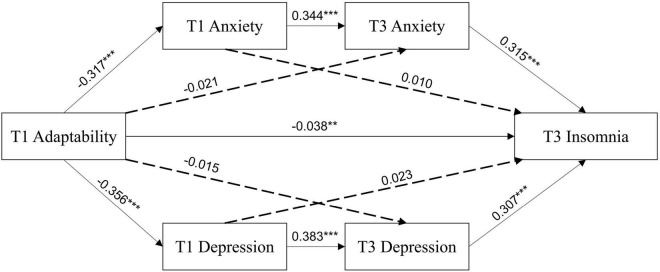
Multiple-mediating test of adaptability, anxiety, depression, and insomnia at T1 and T3. **p < 0.01, ***p < 0.001.

The results of the regression analysis examining variables from T1 and T3 are shown in Figure 3 and Table 5. T1 adaptability significantly negatively predicted T1 anxiety (β = −0.317, *p* < 0.001), T1 depression (β = −0.356, *p* < 0.001), and T3 insomnia (β = −0.038, *p* < 0.01). T1 adaptability did not significantly predict T3 anxiety (β = −0.021, *p* = 0.119) or T3 depression (β = −0.015, *p* = 0.264). T1 anxiety positively predicted T3 anxiety (β = 0.344, *p* < 0.001), but not T3 insomnia (β = 0.010, *p* = 0.620). T1 depression positively predicted T3 depression (β = 0.383, *p* < 0.001), but not T3 insomnia (β = 0.023, *p* = 0.271). A strong regressive path was shown between T3 anxiety and T3 insomnia (β = 0.315, *p* < 0.001) and between T3 depression and T3 insomnia (β = 0.307, *p* < 0.001). The final regression model for insomnia, which included T1 adaptability, T1 anxiety, T3 anxiety, T1 depression, and T3 depression, was highly significant and accounted for 36% of the variability in T3 insomnia.

**TABLE 5:** Regression analysis of variable relationships at T1 and T3 (*n* = 5235).

Dependent variables	Predictors	Model summary				
		*F*	*R* ^2^	β	*SE*	*t*
T1 Anxiety	T1 Adaptability	584.13***	0.10	–0.317	0.016	–24.17
T3 Anxiety	T1 Adaptability	368.25***	0.12	–0.021	0.016	–1.56
	T1 Anxiety			0.344	0.013	25.20
T1 Depression	T1 Adaptability	758.14***	0.13	–0.356	0.016	–27.53
T3 Depression	T1 Adaptability	464.17***	0.15	–0.015	0.016	–1.12
	T1 Depression			0.383	0.013	28.06
T3 Insomnia	T1 Adaptability	599.36***	0.36	–0.038	0.007	–3.18
	T1 Anxiety			0.010	0.010	0.50
	T3 Anxiety			0.315	0.009	18.12
	T1 Depression			0.023	0.010	1.10
	T3 Depression			0.307	0.009	17.41

***p < 0.001.

For the multiple-mediating model of variables from T1 and T3, the direct path from T1 adaptability to T3 insomnia was significant (β = −0.038, 95% CI [−0.037, −0.009]), accounting for 27.74% of the total effect. The chain intermediary effect of T1 and T3 anxiety (β = −0.034, 95% CI [−0.043, −0.027]) was significant, as well as the chain intermediary effect of T1 and T3 depression (β = −0.042, 95% CI [−0.050, −0.034]), indicating a significant chain mediation by both anxiety and depression at two time points. These two paths accounted for 24.82 and 30.66% of the total effect, respectively (see Table 6). T1 adaptability did not indirect impact T3 insomnia via T1 anxiety, T3 anxiety, T1 depression, or T3 depression, as shown in Table 6.

**TABLE 6:** Mediation effects test at T1 and T3.

	Indirect paths (Ind)	Indirect effect			Percentage
		Effect	95% confidence interval		
			BootLLCI	BootULCI	
Ind 1	T1 Adaptability→T1 Anxiety→T3 Insomnia	−0.003	−0.018	0.012	2.19%
Ind 2	T1 Adaptability→T3 Anxiety→T3 Insomnia	−0.007	−0.016	0.003	5.11%
Ind 3	T1 Adaptability→T1 Depression→T3 Insomnia	−0.008	−0.026	0.010	5.84%
Ind 4	T1 Adaptability→T3 Depression→T3 Insomnia	−0.005	−0.014	0.004	3.65%
Ind 5	T1 Adaptability→T1 Anxiety→T3 Anxiety→T3 Insomnia	−0.034	−0.043	−0.027	24.82%
Ind 6	T1 Adaptability→T1 Depression→T3 Depression→T3 Insomnia	−0.042	−0.050	−0.034	30.66%
Sum		−0.099	−0.120	−0.077	72.26%

*Percentage, Percentage of total effect explained.*

## Discussion

In the current study, we tested two multiple mediating models examining the role of adaptability, anxiety, and depression in predicting university students’ insomnia both during the COVID-19 lockdown and a year after the lockdown had begun. Although past studies have shown the key role of adaptability in sleep responses to crises [e.g., as the only significant factor influencing sleep disturbance following an earthquake; (29)], the present study elucidates the mechanisms underlying these relationships and further contributes longitudinal data to this issue. The results demonstrate that adaptability protects individuals from insomnia both directly and indirectly, by decreasing their anxiety and depression.

To examine how self-reported adaptability before a crisis predicts insomnia, we tested two models. Together, they provide consistent results concerning the role of pre-existing adaptability, anxiety, and depression for subsequent anxiety, depression, and insomnia both 6 and 17 months later. The only notable difference between the two models is the significant direct effect from T1 adaptability to T3 insomnia found in the second model. We suspect that students had to adapt to numerous changes over the year—from lockdown to return to school and numerous waves of infection risk—which led to an accentuated effect of this personality variable on insomnia (which, on average, increased from T2 to T3). Based on this intriguing finding, we suggest future research should explore how the impact of adaptability is moderated by the instability of the environment.

Overall, the present study breaks new ground in examining how adaptability—the ability to respond positively to challenging situations and changing demands in one’s environment—impacts sleep disorders. We found that adaptability (as a predisposing factor measured before the onset of the precipitating event) directly affected insomnia 17 months after measurement, and indirectly through both anxiety and depression. This highlights the importance of measuring the impact of adaptability longitudinally, as its effects are subtle, continuous, and best seen over time, as individuals’ adaptive changes in cognition, behavior, and affect in response to the environment produce favorable outcomes over time (58). For example, other research conducted during the pandemic found that students who were able to respond productively to the unexpected shift to remote learning and pandemic restrictions responded to their schoolwork with less anxiety, allowing them to engage more fully in their academic studies (19), and as a result, experience fewer sleeping problems (59). Similarly, positive adaptations to the impact of COVID-19 (e.g., living at home, learning remotely) may have allowed students to feel more hopeful and in control, generating less depression, and in turn, less insomnia (60). Importantly, adaptability continued to impact students as they had to adapt – again—to post-lockdown pandemic life, as seen as its direct and indirect effect on T3 insomnia 17 months after the measurement of adaptability.

Anxiety and depression both appeared heightened during the COVID-19 pandemic, particularly among quarantined individuals (61) and those with greater exposure to the media (11, 62). Information highlighting the health risks for older loved ones (63, 64) and possible negative health consequences even for those who recover (65) stoked legitimate fear, with consequences for mental health and sleep health. Social media use related to the pandemic, for example, was found to be associated with fear of COVID-19 and both directly and indirectly related to insomnia (66). Along with anxiety, experiencing depression, loneliness, and feelings of isolation were also considered hallmarks of the COVID-19 pandemic (67), due to the need to self-isolate or quarantine (and for university students, to complete schoolwork remotely). Thus, anxiety and depression are relevant mediators for the COVID-19 context. The present findings illustrate the importance of examining such mediators when examining predictors of insomnia.

There are also important practical implications of the present work that could be applied by universities and mental health counselors. Fostering adaptability should benefit both students’ mental health and sleep health. In times of upheaval, students need to look at their current cognitions, behaviors, and emotions, and then identify what changes they need to make given the changing circumstances to successfully adapt to the new environment. This greater adaptability should be associated with reduced depression and anxiety, and reduced sleep disturbances. The present study demonstrates that this effect lasts beyond the present moment to even a year following the lockdown.

According to the stress-buffering model (68), in times of significant stress, social connections mitigate the negative effects of stress on well-being. Loneliness and social isolation, on the other hand, are associated with anxiety, depression (69), poorer sleep quality (70, 71) and sleep fragmentation (72), and overall mortality risk (73). For the 30 million college students in China who were quarantined at home, the pandemic created a particularly challenging social context for students’ physical and mental health.

In addition to university students, the COVID-19 pandemic and lockdown was particularly difficult for those with chronic health conditions. Research investigating patients with inflammatory rheumatic diseases (IRD), including rheumatoid arthritis, psoriatic arthritis, axial spondylarthritis, and others, showed a greater level of self-reported concerns and anxiety (74). Other research showed patients with relapsing-remitting multiple sclerosis (RRMS) were more susceptible to the detrimental neuropsychiatric effects of the pandemic and this condition was associated with a higher psychiatric concern, with a report of 48.6% moderate to severe anxiety, 22% moderate to severe depression and 29.6% insomnia by RRMS patients (75). Future research should move beyond the current population of university students to examine the predisposing and precipitating factors associated with insomnia in these populations.

One limitation of the present study was that the psychological assessments were obtained with self-report, which may limit the objectivity of the data. Secondly, to eliminate the possibility of participants remembering (and simply repeating) their responses to the earlier assessment [i.e., memory effects; (76)] as well as to avoid boredom effects during the second assessment, we used different anxiety and depression scales at the second time point (as compared to the first and third). Both the anxiety and depression scales of the SCL-90, as well as the SAS and SDS, are highly reliable and valid measurements used in previous research [e.g., (39, 77)]. As Marcoulides and Grimm (78) demonstrate, when using different measures, outcome variables must be calculated based on a common metric. For this reason, standardized values of these different measures were applied in our analyses [e.g., (79)]. Although this may be perceived a potential limitation for our conclusions, we found similar relationships with these different measures as were observed with identical measures (e.g., T1 anxiety and depression significantly predicted both T2 anxiety and depression and T3 anxiety and depression, respectively, and both contributed to a significant chain mediation of T1 adaptability on T3 insomnia). Third, although the university students came from more than 20 provinces, they all came from China. Future research should replicate this model in other regions of the world.

## Conclusion

The present longitudinal study examined the mechanisms underlying the relationship between adaptability and insomnia. We found that higher levels of pre-pandemic levels of adaptability protected students from insomnia both directly and indirectly, through its negative relationship with anxiety and depression. The present findings add to our understanding of how adaptability enables students to successfully navigate unexpected challenges like COVID-19, by protecting their mental health and sleep quality.

## Data Availability Statement

The datasets presented in this study can be found in online repositories. The names of the repository/repositories and accession number(s) can be found below: The data that support the findings of this study are openly available in [“OSF”] at https://osf.io/vqe94/?view_only=3b5754af106d44f99807367471be7670.

## Ethics Statement

The studies involving human participants were reviewed and approved by the Department of Psychology, Qingdao University. Written informed consent to participate in this study was provided by the participants’ legal guardian/next of kin.

## Author Contributions

KZ and ZM conceived and designed the survey, performed the survey, and contributed to materials and analysis tools. KZ and WC analyzed the data. KZ, ZM, EP-S, WC, YJ, and RJ wrote the manuscript. KZ, EP-S, WC, YJ, and RJ contributed to literature research. All authors contributed to the article and approved the submitted version.

## Conflict of Interest

The authors declare that the research was conducted in the absence of any commercial or financial relationships that could be construed as a potential conflict of interest.

## Publisher’s Note

All claims expressed in this article are solely those of the authors and do not necessarily represent those of their affiliated organizations, or those of the publisher, the editors and the reviewers. Any product that may be evaluated in this article, or claim that may be made by its manufacturer, is not guaranteed or endorsed by the publisher.

## Footnotes

1
https://www.wjx.cn/
